# Prevalence and predictors of viral load non-suppression among adolescents on dolutegravir-based antiretroviral therapy: A cross-sectional study from three urban clinics, Soroti City

**DOI:** 10.1371/journal.pone.0331835

**Published:** 2025-09-09

**Authors:** Connie Nait, Simple Ouma, Saadick Mugerwa Ssentongo, Boniface Oryokot, Abraham Ignatius Oluka, Raymond Kusiima, Victoria Nankabirwa, John Bosco Isunju

**Affiliations:** 1 School of Public Health, College of Health Sciences, Makerere University, Kampala, Uganda; 2 Directorate of Research and Capacity Development, The AIDS Support Organization, Kampala, Uganda; 3 Directorate of Program Management and Capacity Development, AIDS Information Center, Kampala, Uganda; UniCamillus: Saint Camillus International University of Health and Medical Sciences, CAMEROON

## Abstract

**Background:**

Despite advances in HIV care, viral load suppression (VLS) among adolescents living with HIV (ALHIV) in Uganda continue to lag behind that of adults, even with the introduction of dolutegravir (DTG)-based regimens, the Youth and Adolescent Peer Supporter (YAPS) model, and community-based approaches. Understanding factors associated with HIV viral load non-suppression in this population is critical to inform HIV treatment policy. This study assessed the prevalence and predictors of viral load non-suppression among ALHIV aged 10–19 years on DTG-based ART in Soroti City, Uganda.

**Methods:**

We conducted a cross-sectional study among 447 ALHIV attending three urban HIV clinics in Soroti City. Data were abstracted using a structured questionnaire and analyzed in STATA 15.0. Modified Poisson regression with robust error variance was used to identify predictors of viral load non-suppression. Adjusted relative risks (aRR) and 95% confidence intervals (CIs) were reported, with statistical significance set at p ≤ 0.05.

**Results:**

Of the 447 participants, 53.5% were female, with a median age of 16 years (IQR: 14.0–17.6). The majority (72.9%) were from Soroti district and had been on DTG-based ART for a median of 42.5 months (IQR: 37.0–48.0). Most were receiving multi-month dispensing (MMD) (75.2%) and were active in care (98%). The prevalence of viral load non-suppression was 19.2% (86/447). Independent predictors of non-suppression included older age (15–19 vs. 10–14 years) (aRR: 1.69; 95% CI: 1.08–2.67), male sex (aRR: 1.48; 95% CI: 1.05–2.11), prior non-suppression before switching to DTG (aRR: 1.76; 95% CI: 1.19–2.59), use of non-fixed dose DTG regimens (aRR: 2.03; 95% CI: 1.23–3.33), history of poor adherence (aRR: 4.36; 95% CI: 2.05–9.26), and not receiving MMD (aRR: 2.83; 95% CI: 1.93–4.15).

**Conclusion:**

Nearly one in five adolescents on DTG-based ART in Soroti City had viral non-suppression, despite optimized treatment regimens. Targeted interventions−particularly enhanced adherence counseling for older and male adolescents, expanding MMD coverage, and provision of fixed-dose regimens−are urgently needed to improve VLS among ALHIV. These findings underscore the need for adolescent-centered HIV care strategies to close the viral suppression gap and advance progress towards epidemic control.

## Introduction

HIV/AIDS remains one of the greatest public health challenges in the world, with sub-Saharan Africa (SSA) having the biggest burden. An estimated 1.65 million adolescents aged 10–19 years were living with HIV at the end of 2022, of the 39.0 million people living with HIV worldwide [1]. Approximately 140,000 adolescents were newly infected and an estimated 27,000 died of AIDS-related illnesses in the same year [1]. In Uganda, it is estimated that nearly 80,000 adolescents and 1,400,000 adults were living with HIV/AIDS in 2022 [2].

To end HIV as a public health threat by 2030, the Joint United Nations Programme on HIV/AIDS (UNAIDS) set the 95-95-95 targets (diagnosis of 95% of HIV-infected people, provision of treatment for 95% of people diagnosed with HIV, and viral suppression in 95% of treated people). As such, there has been a rapid scale up of ART, use of more effective DTG based regimens and client centered service delivery models among adolescents living with HIV (ALHIV) in many countries in SSA in recent years. Uganda adopted the “test-and-treat” policy among adolescents in 2013 to accelerate epidemic control [3]. However, for epidemic control to be achieved, the ALHIV initiated on ART need to be retained in care and they should attain and sustain HIV viral load suppression.

However, there are several barriers such as the development of side effects, long duration on ART and transition challenges among ALHIV, these remain a big challenge for HIV programs in SSA and form an obstacle to the attainment of the second and third UNAIDS 95-95-95 targets [4]. Additional barriers to retention in care and VL suppression targets include lifestyle challenges such as the absence of medications when away from home, schedule changes and the stigma associated with HIV diagnosis [4]. Strategies to enhance the attainment of retention in care and VL suppression targets include; strengthening of adolescent friendly services, use of simple regimens, and tailoring regimens with the patient’s daily and family activities.

The World Health Organization (WHO) recently approved the integrase strand transfer inhibitors (INSTIs) that include DTG as preferred regimens for treating ALHIV [5]. DTG has interesting pharmacokinetic and pharmacodynamic features such as prolonged intracellular half-life, absence of negative interactions with other anti-retroviral drugs, and potent activity against HIV-1 strains that are resistant to other INSTIs [6]. Its use is currently approved for treatment of CALHIV aged ≥4 weeks and weighing ≥3 kg [7]. The safety and efficacy of DTG among adolescents was supported by the results from the IMPAACT P1093 study [8] and the ODYSSEY clinical trial [9]. However, several questions remain about the rollout of DTG in LMICs, especially in ALHIV where the variations in pharmacogenetics, nutritional status, and other socio-demographic characteristics may significantly affect the effectiveness and safety of the regimen [10]. It has been reported that virological failure was high among patients on DTG born in SSA compared to patients born in other countries. In fact, ALHIV in SSA continue to have lower rates of viral suppression, and higher rates of mortality in comparison to adults [11].

Currently, in Uganda, DTG is being used for the treatment of most ALHIV and has been used since 2018 [12]. Despite its reported potent antiviral activity from clinical trials, program reports at The AIDS Support Organization Center Of Excellence (TASO COE) and Soroti Regional Referral Hospital (SRRH) indicate high levels of viral load non-suppression among ALHIV [13]. Research data on the effectiveness of DTG in program and clinical settings is scant. Similarly, little is known about the predictors of HIV viral load non-suppression among ALHIV on DTG-based ART aged 10–19 years in the Teso region, northeastern, Uganda.

The use of dolutegravir in the treatment of HIV among ALHIV is based on solid clinical trials and experiences of high-income countries (HIC); however, real-world data specifically for Soroti City remains limited. Townsend et al in their study recommended long-term monitoring of the use of integrase inhibitors in adolescent populations to fill evidence gaps [14], Furthermore, a study conducted in Eastern Ethiopia recommended more comprehensive and broader research to explore reasons for virological non-suppression among HIV-positive individuals on dolutegravir-based Antiretroviral treatment [15]. Another study conducted in Southern Tanzania recommended further exploration of characteristics of ALHIV failing on INSTI regimens [16]. This study aimed to understand the predictors of HIV viral load non-suppression among ALHIV on DTG-based ART, as well as the characteristics of ALHIV failing on INSTI regimens. This will enable health providers to effectively select and implement programs and interventions that aim at achieving HIV viral load suppression among ALHIV which will improve their quality of life and also curb onward transmission of HIV.

## Materials and methods

### Study design

This was a cross-sectional study. The data was collected from 1^st^ to 15^th^ July 2024.

### Study setting

The study was conducted at three high-volume ART clinics in Soroti City offering comprehensive HIV/AIDS prevention, care, and treatment services; TASO Soroti Centre of Excellence, Soroti Regional Referral Hospital (SRRH) and Uganda Cares. The 3 clinics are located in Soroti City, Eastern Uganda, approximately 330 km east of Uganda’s capital city, Kampala. By the end of December 2023, 6090, 3200 and 2740 PLHIV including 269, 144 and 105 ALHIV aged 10–19 years were active in care at TASO Soroti, SRRH and Uganda Cares respectively [17]. PLHIV attending these three clinics come from various districts throughout the Teso region and beyond, as far away as Kapelebyong and Kaberamaido districts that are about 80kms from Soroti City.

All the 3 clinics are located in the Soroti Hospital complex with a team of clinicians, adolescent counselors, and social workers who provide HIV care according to MOH guidelines including routine clinical assessment at every visit, viral load testing, and CD4 testing [18]. Viral load testing is done six months after the start of ART and thereafter every six months for children and adolescents to identify those who have unsuppressed VL and are in need of interventions. The caretakers of the unsuppressed children and adolescents are then taken through intensive adherence counselling (IAC) for three months, at least one month apart before a repeat VL is done to determine those failing who are switched to the next line or referred for resistance testing. Children due for VL testing are identified using the electronic system or clinician file recordings. Blood samples (either DBS or plasma) are collected and shipped weekly to central public health laboratories (CPHL) through the SRRH hub.

### Study population

The study population comprised of ALHIV aged 10–19 years on DTG-based ART at the 3 clinics. We included ALHIV aged 10–19 years who had been on DTG-based ART regimens for more than 6 months and were active in care. We excluded ALHIV aged 10–19 years on DTG-based ART for more than 6 months with missing or inconclusive VL results.

### Sample size estimation

Given that the study utilized secondary data that was already collected, we did not estimate the sample size using a formula but instead reviewed the records of all adolescents who had been on DTG-based ART for more than 6 months. We collected data from 458 adolescents who were active in care during the time of our study and met the eligibility criteria.

### Study variables

The **dependent variable** was HIV viral load non-suppression among ALHIV on DTG-based ART aged 10–19 years. HIV viral load non-suppression was defined as; viral load copies that were equal to or greater than 201 copies per milliliter of blood according to Uganda MOH 2022 consolidated guidelines for HIV prevention and treatment.

The most recent viral load was considered.

The **independent variables** were grouped into; Socio-demographic factors that included sex of the adolescent, age, address of the adolescent, and type of residence

Clinical factors that included duration on ART, baseline ARV regimen, current ARV regimen, duration on DTG-based ART, WHO clinical stage, nutrition status, adherence, and co-morbidities, Psychosocial factors that included mental health disorders, Health facility factors that included clinic support groups, facility-based DSD models and Community factors that included community support groups, community-based DSD models. Measurement of all the independent variables and their definitions have been done in a table that has been uploaded in the supplement (S1 Table).

### Data collection tools and procedures

We used a designed data abstraction tool to abstract secondary data from the Uganda Electronic Medical Records (EMR) and patient-level files.

The research assistants (RA) who collected the data were first trained on the study objectives, matters related to adolescents, ART, VL, and the confidentiality required during research, secondary data abstraction, and data triangulation. The RAs sought permission from the facility I/Cs, working with the adolescent focal persons and data clerks of the two facilities, they proceeded to abstract data from the EMR first and then the client files for those variables that were missing in the EMR.

The data collected was de-anonymised whereby unique identifiers (serial numbers) were allocated to the study participants instead of using the facility identifiers.

### Data management

All data extracted into Excel 2019 were reviewed for completeness and those with missing data or incomplete data were completed by triangulation of data sources (EMR and client files). Data was then password-protected to ensure no unauthorized access. Missing data was handled by complete case analysis whereby any observation that had a missing value for the outcome variable was automatically discarded and only complete observations were analyzed.

### Data analysis

Data were then imported into STATA version 15.0 and were checked for uniqueness to get rid of duplicates. Variables were renamed and recoded where necessary for easy handling during analysis, continuous numerical responses were entered as absolute values while categorical responses were coded.

Descriptive statistics were conducted and reported as; mean and standard deviation for continuous variables or else median and interquartile range (IQRs) for the continuous variables with skewed distributions. Categorical variables were reported as frequencies and proportions.

The association between HIV Viral load non-suppression and the categorical variables was determined using the Chi-square or Fisher’s exact test, and associations between treatment success and numerical variables were assessed using the student t-test. The variables significant (p < 0.05) at bivariate analysis were included in the multivariable analysis where modified Poisson regression with robust error variance was conducted to assess association between each independent variable with HIV viral load non-suppression using a stepwise model building.

The results were presented as unadjusted and adjusted relative risk with confidence intervals. The final model was checked for goodness-of-fit using the Hosmer- Lemeshow test, and model diagnostics were conducted using the Area under the ROC curve (AUC).

### Ethical considerations

Ethical clearance for this study was sought from the Makerere School of Public Health Research and Ethics Committee (MakSPH-REC), approval number, 437. Administrative clearances were sought from TASO, SRRH and Uganda Cares management. Since we were collecting secondary data, informed consent/assent was waived off. The data obtained from the records was discreetly handled and anonymity was ensured by keeping data safely under lock and key by the Principal Investigator.

## Results

**Recruitment of study participants.** Of the 458 ALHIV aged 10–19 years, 11 had missing variables of interest, including the most recent viral load, and were dropped. A total of 447 ALHIV were included in the final analysis, as shown S1 Fig

### Socio-demographic characteristics

The median age (IQR) of the study participants was 16 (14–17.6) years with more than half of them in the 15–19 years age category (68%, n = 304), and a slightly higher proportion of females (53.7%). Most participants were from the Soroti district (72.9%, n = 341), and had been on DTG based ART for median (IQR) 42.5 (37–48) months. Nutritional status was generally good, with most participants in the green (normal) category (87%, n = 389) as per the last appointment visit. The most common current regimen was TDF/3TC/DTG (79%, n = 353). The majority were receiving multi-month dispensing (75.2%, n = 336) and were active in care (98%, n = 438), more than half (93.3%, n = 417) were classified as having WHO clinical stage one. The majority (65.3%, n = 292) had a Nevirapine-based regimen as the baseline. They were utilizing various differentiated service delivery models, with the Facility-Based Group model being the most common (66.4%, n = 299) as presented in Table 1 below.

**Table 1:** Socio-demographic characteristics of study participants.

Characteristics	Frequency (N)	Percentage (%)
**Study site**		
Soroti Regional Referral Hospital	158	(35.4)
TASO Soroti	197	(44.1)
Uganda Cares	92	(20.6)
**District of residence**		
Amuria	32	(7.2)
Kumi	3	(0.7)
Ngora	15	(3.4)
Soroti	341	(72.9)
Kaberamaido	11	(2.5)
Katakwi	11	(2.5)
Serere	34	(7.6)
Age in completed years: Median (IQR)	16 (14-17.6)	
**Age groups**		
10-14	143	(32)
15-19	304	(68)
DTG duration (months): median (IQR)	42.5 (37-48)	
**Sex**		
Male	208	(46.5)
Female	239	(53.5)
**Nutrition status** ^*^		
Normal	389	(87)
MAM	46	(10.3)
SAM	12	(2.7
**WHO staging**		
WHO staging 1	408	(91.3)
WHO staging 2	29	(6.5)
WHO staging 3	7	(1.6)
WHO staging 4	3	(0.7)
**Baseline ART Regimen**		
DTG based	32	(7.2)
Efavirenz based	106	(23.7)
Nevirapine based	292	(65.3)
Protease Inhibitor based	17	(3.8)
**Type of Dispensing**		
Multi-month dispensing (MMD)	336	(75.2)
No MMD	111	(24.8
**Current ART regimen**		
TDF/3TC/DTG	353	(79)
ABC/3TC/DTG	68	(15.2)
Non fixed dose DTG regimens	26	(5.8)
**Current ART status**		
Active in care	438	(98)
Lost to follow-up	9	(2)
**Current DSDM model** ^*^		
FBG	299	(66.4)
FTDR	57	(12.8)
FBIM	56	(12.5)
CCLAD	20	(4.5)
CDDP	15	(3.4)
**TB suspect**		
No signs of TB	275	(61.5)
TB suspect	172	(38.5)
**CD4 done**		
No	248	(55.5)
Yes	199	(44.5)
**CD4 done (n = 199)**		
< 200	145	(72.9)
≥ 200	54	(27.1)
**Adherence**		
Good	387	(86.6)
Fair	44	(9.8)
Poor	16	(3.6)
**Viral before DTG**		
Non-suppressed	91	(20.4)
Suppressed	284	(63.6)
Unknown	72	(16.1)

* MAM = Moderate acute malnutrition, SAM = Severe acute malnutrition

* DSMD = Differentiated Service Delivery Model, FBG = Facility Based Group, FTDR = Fast track drug refill, FBIM = Facility Based Individual Management, CCLAD = Community Client Led ART Delivery, CDDP = Community Drug Distribution Point.

Nonfixed dose DTG regimens: AZT/3TC/DTG, TDF/3TC/DTG/DRVr, ABC/3TC/DTG/DRV

### Prevalence of HIV viral load non-suppression

The prevalence of HIV virologic non-suppression was 19.2% (95% CI: 15.8–23.2). By study site, TASO Soroti had the highest proportion of virologic non-suppression at 25.89% (51/197) followed by Uganda Cares at 22.83% (21/92). In comparison, Soroti Regional Referral Hospital had the lowest rate of virologic non-suppression at 8.86% (14/158).

### Predictors of HIV viral load non-suppression

From Table 2 below, several factors were found to be associated with HIV virologic non-suppression; study sites, Age group, sex, multi-month dispensing, current ART regimen, being on fixed-dose combination, Current DSD model, being a presumptive TB, adherence to ART, and viral load suppression status before DTG. Virologic non-suppression varied by study site (P < 0.001). Adolescents aged 15–19 had significantly higher non-suppression than those aged 10–14 (22% vs 13.3%: P = 0.029). Males were more likely to be non-suppressed as compared to females (24.6% vs 14.5%: P < 0.004). The non-suppression was higher among clients not on multi-month dispensing (MDD) (39.6%) as compared to those on MDD (39.6% vs 12.5%: P < 0.001). The non-suppression was varied by regimen (P < 0.001) and not being on fixed dose combination as compared to being on fixed dose (46.7% vs 17.3%: P < 0.001). Individuals without TB signs and symptoms had higher non-suppression than the presumptive TB (25.1% vs 9.9%: P < 0.001), having a poor adherence was associated with non-suppression compared to good adherence (62.5% vs 16.3%: P < 0.001), virologic non suppression varied by viral load suppression status before DTG initiation (P < 0.001) as shown in Table 2.

**Table 2:** Bivariable analysis of the factors associated with virologic non-suppression in Soroti City.

Variables		Non suppressed		p-value
		No (n = 361,80.8%)		Yes (n = 86, 19.2%)
**Study site**				
Soroti Regional Referral Hospital	158 (35.4)	144 (91.1)	14 (8.9)	**0.001** ^*^
TASO Soroti	197 (44.1)	146 (74.1)	51 (25.9)	
Uganda Cares	92 (20.6)	71 (77.2)	21 (22.8)	
**District of residence**				
Amuria	32 (7.2)	25 (6.9)	7 (8.1)	0.878
Kumi	3 (0.7)	2 (0.6)	1 (1.2)	
Ngora	15 (3.4)	11 (3.1)	4 (4.7)	
Soroti	341 (72.9)	278 (77)	63 (73.3)	
Kaberamaido	11 (2.5)	8 (2.2)	3 (3.5)	
Katakwi	11 (2.5)	10 (2.5)	3 (3.5)	
Serere	34 (7.6)	27 (7.5)	7 (8.1)	
**Age groups**				
10-14	143 (32)	124 (86.7)	19 (13.3)	**0.029** ^*^
15-19	304 (68)	237 (78)	67 (22)	
**Sex**				
Female	239 (53.5)	205 (85.8)	34 (14.2)	**0.004** ^*^
Male	208 (46.5)	156 (75)	52 (25)	
**Nutrition status**				
Normal	389 (87)	317 (81.5)	72 (18.5)	0.396
MAM	46 (10.3)	36 (78.3)	10 (21.7)	
SAM	12 (2.7)	8 (66.7)	4 (33.3)	
**WHO staging**				
WHO staging 1	408 (91.3)	330 (80.9)	78 (19.1)	0.911
WHO staging 2	29 (6.5)	23 (79.3)	6 (20.1)	
WHO staging 3	7 (1.6)	6 (85.7)	1 (14.3)	
WHO staging 4	3 (0.7)	2 (66.7)	1 (33.3)	
**Baseline ART Regimen**				
DTG based	32 (7.2)	29 (90.6)	3 (9.4)	0.194
Efavirenz based	106 (23.7)	86 (81.1)	20 (18.9)	
Nevirapine based	292 (65.3)	230 (78.8)	62 (21.2)	
Protease Inhibitor based	17 (3.8)	16 (94.1)	1 (5.9)	
**Multi-month dispensing (MMD)**				
Yes	336 (75.2)	294 (87.5)	42 (12.5)	**0.001** ^*^
No	111 (24.8)	67 (60.4)	44 (39.6)	
**Current ART regimen**				
TDF/3TC/DTG	353 (79)	294 (83.3)	59 (16.7)	**0.001** ^*^
ABC/3TC/DTG	68 (15.2)	54 (79.4)	14 (20.6)	
Non fixed dose DTG regimens	26 (5.8)	13 (50)	13 (50)	
**Current ART status**				
Active in care	438 (98)	206 (81)	83 (19)	0.279
Lost to follow-up	9 (2)	6 (66.7)	3 (33.3)	
**Current DSDM model** ^*^				
FBG	299 (66.4)	243 (81.3)	56 (18.7)	**0.001** ^*^
FTDR	57 (12.8)	55 (96.5)	2 (3.5)	
FBIM	56 (12.5)	37 (66.1)	19 (33.9)	
CCLAD	20 (4.5)	15 (75)	5 (25)	
CDDP	15 (3.4)	11 (73.3)	4 (26.7)	
**TB status**				
No signs of TB	275 (61.5)	206 (74.9)	69 (25.1)	**0.001** ^*^
Presumptive TB	172 (38.5)	155 (90.1)	90 (9.9)	
**CD4 done**				
No	248 (55.5)	220 (80.7)	48 (19.4)	0.945
Yes	199 (44.6)	161 (80.8)	38 (19.2)	
**Adherence**				
Fair	387 (86.6)	31 (70.5)	13 (29.6)	**0.001** ^*^
Poor	44 (9.8)	6 (37.5)	10 (62.5)	
Good	16 (3.6)	324 (83.7)	63 (16.3)	
**Viral suppression before DTG**				
Non-suppressed	91 (20.4)	60 (65.9)	31 (34.1)	**0.001** ^*^
Suppressed	284 (64.6)	245 (86.3)	39 (13.7)	
Unknown	72 (16.1)	56 (77.8)	16 (22.2)	

* FDC = Fixed dose combination, Non-FDC DTG regimens include: AZT/3TC/DTG, DRVr/DTG, TDF/3TC/DTG/DRVr

### Multivariable analysis investigating the factors associated with HIV virologic non-suppression among study participants

The multivariable analysis identified several key associated factors with HIV virologic non-suppression. Independent predictors of non-suppression included older age (15–19 vs. 10–14 years) (aRR: 1.69; 95% CI: 1.08–2.67), male sex (aRR: 1.48; 95% CI: 1.05–2.11), prior non-suppression before initiating DTG (aRR: 1.76; 95% CI: 1.19–2.59), use of non-fixed dose DTG regimens (aRR: 2.03; 95% CI: 1.23–3.33), history of poor adherence (aRR: 4.36; 95% CI: 2.05–9.26), and not receiving MMD (aRR: 2.83; 95% CI: 1.93–4.15) as shown in Table 3 below

**Table 3:** Multivariable analysis of the factors associated with virologic non-suppression in Soroti City.

Variable	Crude Relative Risk (CI 95%)	p-value	Adjusted Relative Risk (CI 95%)	p-value
**Age categories**				
10–14	**Reference**			
15–19	1.65 (1.04-2.65)	0.035	1.69 (1.08-2.67)	0.022*
**Sex at birth**				
Female	**Reference**			
Male	1.68 (1.14-2.49)	0.008	1.48 (1.05-2.11)	0.027*
**Study site**				
SRRH	**Reference**			
TASO Soroti	2.92 (1.67-5.08)	0.001	2.14 (0.67-6.89)	0.202
Uganda Cares	2.57 (1.37-4.81)	0.003	2.74 (0.81-9.27)	0.104
**Viral before DTG**				
Suppressed	**Reference**			
Non-suppressed	2.48 (1.64-3.73)	0.001	1.76 (1.19-2.59)	0.004*
Unknown	1.61 (0.96-2.72)	0.071	1.28 (0.72-2.28)	0.39
Current ART regimen*				
TDF/3TC/DTG	**Reference**			
ABC/3TC/DTG	1.23 (0.73-2.07)	0.434	1.28 (0.75-2.19)	0.361
Non fixed dose DTG regimens	2.99 (1.91-4.69)	0.001	2.03 (1.23-3.33)	0.005*
**Adherence to ART**				
Fair	**Reference**			
Poor	2.11 (1.16-3.82)	0.013	4.36 (2.05-9.26)	0.001*
Good	0.55 (0.33-0.92)	0.022	0.76 (0.45-1.27)	0.307
**Multi-month dispensing**				
Yes	**Reference**			
No	3.17 (2.20-4.56)	**0.001**	2.83 (1.92-4.13)	0.001*
**TB status**				
No signs of TB	**Reference**			
Presumptive TB	0.39 (0.23-0.64)	**0.001**	1.25 (0.44-3.54)	0.665
Current DSDM model*				
CCLAD	**Reference**			
CDDP	1.06 (0.34-3.31)	0,911	0.96 (0.35-2.61)	0.937
FBG	0.74 (0.33-1.66)	0.477	0.95 (0.49-1.85)	0.891
FTDR	0.14 (0.03-0.66)	**0.014**	0.23 (0.04-1.13)	0.071
FBIM	1.35 (0.58-3.15)	0.478	1.16 (0.55-2.44)	0.695

## Discussion

The study found that the proportion of adolescents on ***Dolutegravir-Based Antiretroviral Therapy*** with a recently non-suppressed viral load was high at 19.2%. Adolescents in the age group of 15–19 years, being a male, having a history of VL non suppression before initiating DTG, poor adherence, being on non-fixed dose combination DTG regimens and not receiving MMD significantly increased the chance of being non-suppressed.

In this study we found that the proportion of adolescents with a recently non-suppressed viral load was 19.2%. This was higher than the UNAIDS target of 5% non-suppressed [19]. A previous study on HIV viral load suppression rate among adolescents in Kenya reported similar findings (Viral non-suppression = 20%) [20]. However, this viral load non-suppression rate is lower than that reported in two previous Zimbabwe studies which were at 35.1% and 46.8% [21,22]. In this study, we found that about 96% of adolescents at our study sites are receiving DTG-based ART now, this is a possible reason for lower non-suppression as compared to the two previous studies. A comparable study conducted among children and adolescents living with HIV in Tanzania found high viral suppression rates of 91.64% [23]. The figure of our viral load non-suppression rate is slightly lower than that of a similar study conducted among adults in the DRC that found that 27.2% of the participants had a VL of >50 copies per ml [24]. High viral load non-suppression could have been due to; poor medication adherence, and missed clinic appointments which resulted in intermittent access to ART, drug-resistant mutations, and adverse drug effects. Similarly, these adolescents are at substantial risk of stigma and its harmful effects on adherence and viral load outcomes. Comparable studies across African countries have also found that being an adolescent is a key predictor of failure to achieve VL suppression [25]. A previous retrospective study found that the availability of youth-friendly services plus trained providers was associated with increased rates of virological suppression [26]. Similarly, peer-based strategies are now recommended at HIV clinics by WHO and are showing promising virological results.

This study found that male adolescents had a high likelihood of virologic non-suppression compared to females. This could be because most males report to health facilities at advanced stages of the disease which contributes to interruption of treatment. Boys and men also have lower engagement in psychosocial support services. This is consistent with previous findings where being a male was associated with HIV viral load non-suppression and virologic failure in South Africa [27], Zimbabwe [21], and Kenya [28]. Among adults in SSA, there is good evidence of poorer viral suppression among male individuals [29–31]. This was initially thought to be a result of late presentation, poor adherence, and higher substance abuse. However, there is emerging evidence of possible biological differences in drug metabolism [32]. However, two studies conducted in Ghana [33] and Tanzania [34] showed contrary findings that females were 2.5 times more likely to have virological non-suppression as compared to males. While other studies have found no association between sex and virological non-suppression [35], Njom et al. think that the role of gender in virological suppression could be biological [36]. Therefore, the relationship between virological suppression and gender is inconclusive and requires further studies.

Our study found that adolescents on non-fixed dose combination DTG regimens had higher odds of virologic non-suppression as compared to adolescents on TDF/3TC/DTG. Our findings are consistent with a previous study done in Uganda and Tanzania where high VL suppression rates were found among those on TDF/3TC/DTG and ABC/3TC/DTG, this could have been due to once-a-day dosing compared to AZT/3TC/DTG and third-line regimens that are usually taken twice daily and have higher pill burdens [37–39]. Previous studies have reported that a high pill burden is associated with poor adherence, which in turn is linked to lower rates of viral load suppression [40,41]. A majority of these regimens are third-line ART implying that the adolescent has failed both first-line and second-line regimens, there’s therefore a possibility that the adolescent has developed drug resistance and mutations across most of the drug classes rendering the drugs ineffective to suppress the viral load.

In this study we found adherence as a strong predictor of virologic non-suppression. Those with poor adherence had significantly higher odds of non-suppression versus those with good adherence. This is in agreement with the previous findings where poor adherence was reported to be associated with virologic failure [21,42].This is because adherence maintains an optimal drug level and allows the drug to achieve the desired effects of suppressing viral replication thus promoting immune recovery. In a recent study however, intensive adherence interventions resulted in suppression among 23% of children and adolescents [43], compared with 70% suppression in adults, which may indicate a higher likelihood of resistant viruses in children and adolescents emphasizing the need to achieve and sustain good adherence in the population [44].

Adolescents who were not receiving MMD were more likely to be non-suppressed as compared to those who were enrolled in MMD. Our findings are consistent with earlier findings in Nigeria, and Uganda that MMD increased the VLS rate [45–47]. However this was contrary to the findings in South Africa were VLS was similar among ART adherence club members who received ART for six months with annual clinical consultations compared with those receiving ART for two months [48]. This is because an adolescent who is not receiving MMD is expected to have a clinical encounter every month, which could possibly lead to missed clinic appointments and then frequent drug interruptions and consequently non-viral suppression.

This study also found that adolescents in the age group of 15–19 years were more likely to be non-suppressed as compared to their counterparts in the age group of 10–14 years. This is contrary to two previous studies conducted in Uganda and Tanzania that found that HIV viral load non-suppression was more common among children and lower adolescents compared to the older ones [16,49]. Related findings in Swaziland also revealed that pre-adolescents (10–14) were more likely to remain non-suppressed at retesting following completion of IAC [35]. HIV viral load non-suppression among the older adolescents (15–19) years could have been because of negative peer pressure, stigma, and the discovery of sexuality, these affect their ability to access quality HIV care and make proper health decisions.

This study further found that adolescents who had a history of VL non-suppression before initiation on DTG based ART were more likely to be non-suppressed as compared to those who had a suppressed VL. This is consistent with findings of a previous study that found that having an initial VL between 1000 and 50,000 copies/ml was a key predictor of VL non-suppression [25]. Having a history of VL non-suppression could imply the development of drug resistance and mutations across the different ART drug classes rendering the drugs ineffective to suppress the viral load.

### Limitations of the study

The study used secondary data, conclusions could have been limited by issues of data quality and incomplete values which were mitigated with triangulation of data from EMR and patient records. General HIV drug resistance testing was unavailable for this study, preventing any conclusions about VL suppression due to drug failure. Additionally, this study’s method for measuring adherence by utilizing clinic records that were captured by self-reports likely underestimates the true rate of non-adherence.

However, this study had notable strengths of being done in ART clinics with the greatest number of ALHIV active in care in the region and including all ALHIV in care making the findings generalizable. This study adds to the limited literature on viral load suppression among the ALHIV on a DTG-based regimen and complements Uganda and PEPFAR efforts to optimize the ALHIV on the efficacious DTG-based regimen to achieve the UNAIDS target by 2030.

## Conclusion

Our study demonstrates high rates of viral non-suppression among the adolescent population living with HIV, which was associated with the adolescents in the age group of 15−19 years, male sex, having a history of VL non-suppression before initiating DTG, being on non-fixed dose combination DTG regimens, having poor adherence, and not receiving MMD. Therefore, there is a need to optimize adherence strategies among ALHIV especially the male and older adolescents, implement routine HIV resistance testing and scale up fixed-dose DTG regimens to improve VL suppression which will improve treatment outcomes for adolescents. HIV programs then need to design targeted age and gender specific care and treatment policies, programs, and interventions, or to modify the existing ones, to improve VL suppression among adolescents living with HIV on DTG-based ART, to achieve the 95-95-95 targets by 2030.

## Supporting information

S1 Data Dataset on the prevalence and predictors of HIV viral load non-suppression (excel). (CSV)

S2 Questionnaire Questionnaire on the prevalence and predictors of HIV viral load non-suppression.
(PDF)

S3 Strobe Checklist Checklist of items that should be included in reports of observational studies.
(DOCX)

S1 Table Definitions and measurement of independent variables. (PDF)

S1 Fig Study flow. (TIF)
