# Supplementary material for: Prevalence and predictors of viral load non-suppression among adolescents on dolutegravir-based antiretroviral therapy: A cross-sectional study from three urban clinics, Soroti City
Source: PLoS One. 2025 Sep 9;20(9):e0331835. doi: 10.1371/journal.pone.0331835 (PMC12419619; doi:10.1371/journal.pone.0331835)
Supplement: S2 Questionnaire — Questionnaire on the prevalence and predictors of HIV viral load non-suppression. (PDF) [file pone.0331835.s002.pdf]

	**<u>SECTION 1: IDENTIFICATION SECTION</u>**		
1.1	District: …………………………....	1.5	Sub County……………………………
1.2	Village: ……………….……………	1.6	Interviewer name: ………………...
1.3	Extraction Date: ……………………	1.7	Tribe: ………………………………….
1.4	Patient I.D: …………………………	1.8	Health facility: ……………………....
	**SECTION 2: HIV VIRAL LOAD STATUS**		
2.1	The most recent viral load results of the participant		
2.2	Date of the most recent viral load		DD …/ …MM …/ …YYYY …
2.3	Viral load results prior to being switched/transitioned to DTG		
	**PREDICTORS OF HIV VIRAL LOAD NON SUPPRESSION AMONG ALHIV ON DTG-BASED ART**		
	**<u>SECTION 3: SOCIO-DEMOGRAPHIC FACTORS</u>**		
3.1	Sex of the adolescent	1=Male 2=Female	
3.2	Current age		
3.3	Date of Birth:	DD…/…MM…/…. YYYY….	
3.4	Age at Registration into care	……………………	
3.5	Age of the child at initiation of ART	…………………..	
3.6	Residence	1=Urban 2=Rural	
	**<u>SECTION 4: CLINICAL FACTORS</u>**		
4.1	Type of ART regimen at initiation	0=EFV based 1=NVP 2=PI based 3=DTG based	
4.2	Duration on DTG-based ART (in months)		
4.3	Name of the current ART regimen	…………………..	
4.4	Number of ARV pills in the regimen the participant is on	1=One pill/day, 2=more than one pill/day	
4.5	CD4 count before initiation on ART (baseline CD4 count)	0=CD4<250cells/ml, 1=CD4 250<350 cells/ml, 2=CD4 350<500cells/ml, 3=CD4 ≥ 500cells/ml, 4=unknown	

4.6	WHO stage at initiation of ART	0=stage I, 1=stage II, 2=stage III, 3=stage IV, 4=unknown
4.7	Current WHO stage	0=stage I, 1=stage II, 2=stage III, 3=stage IV, 4=unknown
4.8	Current nutrition status	0=Underweight 2=Normal 3=Overweight 4=Obese
4.9	Recorded percentage adherence in the last appointment visit	1=Good 2=Fair 3=Poor
4.10	Receiving drugs for 3 or more months	1=Yes 2=No
4.11	TB status	1=Negative 2=TB suspect 3=Positive on TB treatment
4.12	Patient current status	1=Active 2=Dead 3=Loss to follow up
	**SECTION 5: PSYCHOSOCIAL FACTORS**	
5.1	Diagnosis and treatment for a mental health disorder before	1=Yes 2=No
5.2	Has the Adolescent been disclosed to	1=Yes 2=No
	**SECTION 6: HEALTH FACILITY FACTORS**	
4.1	Intensive Adherence Counselling (IAC) done	1=Yes 2=No
4.2	Does the child/adolescent belong to a facility-based DSD model (FBIM, FBG, FTDR)	1=FBIM 2=FBG 3=FTDR
	**SECTION 7: COMMUNITY FACTORS**	
7.1	Does the child/adolescent belong to a community-based DSD model (CDDP, CCLAD)	1=CDDP 2=CCLAD
