# Supplementary material for: Prevalence and predictors of viral load non-suppression among adolescents on dolutegravir-based antiretroviral therapy: A cross-sectional study from three urban clinics, Soroti City
Source: PLoS One. 2025 Sep 9;20(9):e0331835. doi: 10.1371/journal.pone.0331835 (PMC12419619; doi:10.1371/journal.pone.0331835)
Supplement: S1 Table — (PDF) [file pone.0331835.s004.pdf]

Independent variables; definition and measurement

Independent variable	Definition and measurement of variable	Source of data
Sex	This was measured as a categorical variable: male or female	EMR
Age	This was measured as a continuous variable in completed years	EMR
Address	This was the district of residence of the adolescent as indicated in the medical records	Client files
Type of residence	This was categorized as urban or rural as reflected in the address (urban residence were those from within cities and towns of districts while rural residence were those from distant villages of the districts)	Client files
Duration on ART	This was measured as a continuous variable in completed months from the time of data collection to the time of initiating baseline ART	EMR
Current ART regimen	This was measured as a categorical variable, categorized as a fixed-dose combination and non-fixed dose combination	EMR
Duration on DTG based ART	This was measured as a continuous variable in completed months from the time of collecting data to the time of initiating DTG based ART	EMR
WHO stage	This was measured as a categorical variable as stage I, stage II, stage III, stage IV and unknown stage.	EMR and client files
Adherence	Adherence data was collected from medical records as per the last clinic visit, which was measured as a self-report from the adolescents and/or the caretakers. Presented as a categorical variable measured according to Uganda MOH 2022 consolidated guidelines for HIV prevention and control where an adolescent missing less than 2 doses in the last month was considered to have good adherence (≥95%), 2-5 doses with fair adherence (85-95%), and > 5 doses with poor adherence (<85%)	Client files
CD4 count	This was the most recent documented CD4 count measured as a continuous variable	EMR and client files
Nutritional status	This was measured as a categorical variable and categorized as normal, MAM (Moderate Acute Malnutrition), and SAM (Severe Acute Malnutrition) based on the latest recorded color codes where green represented normal, yellow represented MAM and red represented SAM	EMR
TB status	This was measured as a categorical variable categorized into no sign or symptom of TB and presumptive TB/TB suspect, this was based on the recorded TB status as per the client's last appointment visit.	Client files
Type of dispensing	This was measured as a categorical variable, categorized as multi month dispensing, no multi month dispensing and missing	EMR
Current ART status	This was measured as a categorical variable as active in care or lost to follow up	EMR

DSD models	These are differentiated service delivery models (DSD models) both at the facility and in the community, categorized into facility based and community based models	EMR
