# Supplementary figures and images for: Prevalence and predictors of viral load non-suppression among adolescents on dolutegravir-based antiretroviral therapy: A cross-sectional study from three urban clinics, Soroti City
Source: PLoS One. 2025 Sep 9;20(9):e0331835. doi: 10.1371/journal.pone.0331835 (PMC12419619; doi:10.1371/journal.pone.0331835)

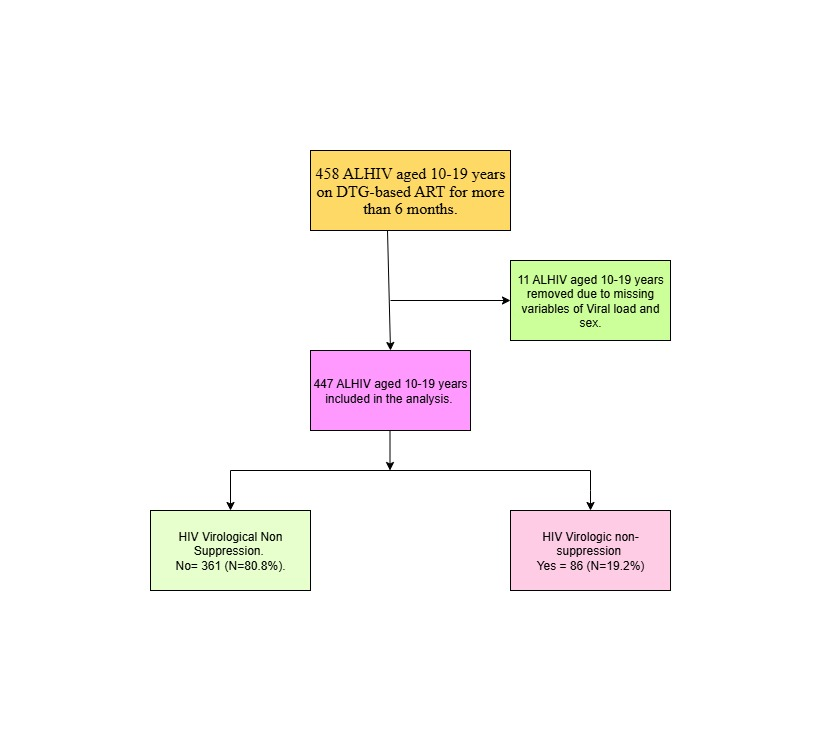

Supplement: S1 Fig — (TIF) [file pone.0331835.s005.tif]
